# Murine CLCA5 is uniquely expressed in distinct niches of airway epithelial cells

**DOI:** 10.1007/s00418-014-1279-x

**Published:** 2014-09-12

**Authors:** Kristina Dietert, Lars Mundhenk, Nancy A. Erickson, Katrin Reppe, Andreas C. Hocke, Wolfgang Kummer, Martin Witzenrath, Achim D. Gruber

**Affiliations:** 1 Department of Veterinary Pathology, Freie Universität Berlin, Berlin, Germany; 2 Department of Infectious Diseases and Pulmonary Medicine, Charité – Universitätsmedizin Berlin, Berlin, Germany; 3 Institute of Anatomy and Cell Biology, Justus-Liebig University, Giessen, Germany; 4 German Center of Lung Research, Giessen, Germany

**Keywords:** Airway epithelial cell, Murine lung, mCLCA3, Mucus cell metaplasia, Translational medicine

## Abstract

The murine mCLCA5 protein is a member of the chloride channel regulators, calcium-activated (CLCA) family and is suspected to play a role in airway mucus cell differentiation. Although mCLCA5 mRNA was previously found in total lung extracts, the expressing cells and functions in the naive murine respiratory tract are unknown. Therefore, mCLCA5 protein expression was identified by immunohistochemistry and confocal laser scanning microscopy using entire lung sections of naive mice. Moreover, we determined mRNA levels of functionally related genes (mClca3, mClca5, Muc5ac and Muc5b) and quantified mCLCA5-, mCLCA3- and CC10-positive cells and periodic acid-Schiff-positive mucus cells in naive, PBS-treated or *Staphylococcus aureus*-infected mice. We also investigated mCLCA5 protein expression in *Streptococcus pneumoniae* and influenza virus lung infection models. Finally, we determined species-specific differences in the expression patterns of the murine mCLCA5 and its human and porcine orthologs, hCLCA2 and pCLCA2. The mCLCA5 protein is uniquely expressed in highly select bronchial epithelial cells and submucosal glands in naive mice, consistent with anatomical locations of progenitor cell niches. Under conditions of challenge (PBS, *S. aureus*, *S. pneumoniae*, influenza virus), mRNA and protein expression strongly declined with protein recovery only in models retaining intact epithelial cells. In contrast to mice, human and porcine bronchial epithelial cells do not express their respective mCLCA5 orthologs and submucosal glands had fewer expressing cells, indicative of fundamental differences in mice versus humans and pigs.

## Introduction

mCLCA5 is a murine member of the chloride channel regulators, calcium-activated (CLCA) protein family which has been linked to inflammatory airway diseases with increased mucus production such as asthma, cystic fibrosis and chronic obstructive pulmonary disease (Brouillard et al. 2005; Hegab et al. 2004; Kamada et al. 2004). It has also been hypothesized that CLCA proteins act as extracellular signaling molecules, transforming airway mucus precursor cells to mature mucus cells (Patel et al. 2009) or, as growing evidence suggests, modulating the innate immune response (Dietert et al. 2014; Long et al. 2006; Zhang and He 2010), pointing toward a pleiotropic function of these proteins (Patel et al. 2009).

The human CLCA1 (hCLCA1), expressed in mucus cells of the respiratory tract (Gibson et al. 2005), is known to regulate mucus cell metaplasia by inducing mucus gene transcription via a downstream mitogen-activated protein kinase (MAPK)-13 signaling pathway (Alevy et al. 2012). In the mouse lung, overexpression of mCLCA3, the murine ortholog of hCLCA1, also induces mucus cell metaplasia; however, mClca3 knockout mice do not show a corresponding phenotype (Patel et al. 2006). Loss of mCLCA3 in these mice has been discussed to be compensated by increased mCLCA5 expression in experimentally induced mucus cell metaplasia in vitro and in vivo (Alevy et al. 2012; Mundhenk et al. 2012; Patel et al. 2006). It has consequently been speculated that the two proteins may have a redundant function in the respiratory tract, which is also supported by the observation that gene transfer with a vector-encoding mClca5 also induces airway mucus production (Patel et al. 2006).

Interestingly, mCLCA5 mRNA has been detected in various tissues, including the respiratory tract of naive mice in which neither its expressing cell type nor its protein has been observed so far (Braun et al. 2010). Instead, mCLCA5 protein was only discovered after Th2-induced airway inflammation (Mundhenk et al. 2012). In unchallenged mice, the protein has only been detected outside the respiratory tract, specifically in late differentiated keratinocytes of all stratified squamous epithelial granular layers throughout the body with a proposed function in growth arrest and maturation of squamous epithelial cells (Braun et al. 2010). In contrast to mCLCA5, the human orthologous hCLCA2 protein appears to be expressed in basal epithelial cells of stratified epithelia, with a proposed role in stratification and basal cell-basement membrane adhesion (Carter et al. 1990; Connon et al. 2004, 2005). However, it has never been detected in airway epithelial cells and, in contrast to the murine mCLCA5, is not overexpressed following induction of mucus cell metaplasia (Alevy et al. 2012). It has been argued that due to these differences between murine and human CLCA orthologs, the value of mouse models for mucus cell metaplasia in translational medicine is questionable. Functional studies on mucus cell differentiation are still lacking, but it has been shown that the expression pattern of pCLCA1, the porcine ortholog to hCLCA1 and to mCLCA3, is virtually identical with that of hCLCA1, supporting the pig as the favored translational model (Plog et al. 2009).

Porcine pCLCA2, the ortholog of hCLCA2 and mCLCA5, is also expressed in airways on mRNA level, but the expressing cell type is still unknown (Plog et al. 2012b). Similarly to mCLCA5, its protein has, so far, only been found in mature keratinocytes of the epidermis and in the inner root sheath of hair follicles (Plog et al. 2012b).

We hypothesized that mCLCA5 protein is expressed in highly select areas of the naive murine lung, since it has only been found on the mRNA level in total lung extracts so far. We further speculated that its expression pattern may be different from that of human and porcine orthologs since the expressional behaviors differ between the murine and the human orthologs following induction of mucus cell metaplasia (Alevy et al. 2012).

Consequently, we systematically characterized the protein expression pattern of mCLCA5 on entire mouse lung sections by immunohistochemistry and localized the protein by confocal laser scanning immunofluorescence microscopy and immunohistochemical double staining for specific cell markers. Since growing evidence additionally suggests a modulating role of CLCA proteins in innate immune response (Dietert et al. 2014; Long et al. 2006; Zhang and He 2010), we determined lung mRNA expression levels of selected genes of interest, including mClca3, mClca5, Muc5ac and Muc5b and quantified cells expressing mCLCA5, mCLCA3 and club (formerly Clara) cell protein CC10 as well as periodic acid-Schiff (PAS)-positive mucus cells from PBS-treated or *Staphylococcus aureus* (*S. aureus*)-infected mice in comparison with naive controls. We further investigated the course of mCLCA5 protein expression in two other lung infection models, *Streptococcus pneumoniae* (*S. pneumoniae*) and influenza virus. To determine possible species-specific differences, we compared the expression pattern of murine mCLCA5 with those of its human and porcine orthologs, hCLCA2 and pCLCA2.

## Materials and methods

### Naive mice and tissue processing

Naive female C57BL/6J wild-type mice, aged 8–9 weeks and weighing 18–20 g, were housed in individually ventilated cages under SPF conditions with a room temperature of 22 ± 2 °C and a relative humidity of 45–65 %. A 12-h light/dark cycle was maintained, and the animals had unlimited access to standard pelleted food and tap water. For experimental procedures, mice were anesthetized each by intraperitoneal injection of premixed ketamine (3.2 mg) and xylazine (1.5 mg) and sacrificed by exsanguination via the caudal *Vena cava*.

For lung tissue processing, whole lungs with tracheas were carefully removed, immersion fixed in 4 % formalin, pH 7.0, for up to 48 h, and subsequently embedded in paraffin. Multiple sections were cut from serial levels of the lung to ensure that the trachea and the complete bronchial stem including its branching points were available for systematic investigation.

### Lung tissue of mouse models, pigs and humans

Similarly processed, formalin-fixed and paraffin-embedded (FFPE) lung tissues or snap-frozen lungs from PBS-treated, *S. aureus*-, *S. pneumoniae*- or influenza virus-infected mice from previous studies (Dames et al. 2014; Dietert et al. 2014; Reppe et al. 2009) were used. Samples from human tracheas and lungs, obtained from body donors corpses at the Institute of Anatomy and Cell Biology, Justus-Liebig University, Giessen, Germany, as well as from healthy porcine tracheas and lungs, taken from the routine necropsy pool of the Department of Veterinary Pathology, Freie Universität Berlin, Germany, were also fixed in 4 % buffered formalin and embedded in paraffin.

### RNA isolation and quantitative RT-PCR

Total RNA was isolated from snap-frozen, murine lungs using the Nucleo Spin RNA/Protein isolation Kit (Macherey–Nagel, Düren, Germany), quality checked and quantified using the NanoDrop ND-100 Spectrophotometer (Peqlab, Wilmington, USA). Transcript expression levels of murine Clca3, Clca5, Muc5ac and Muc5b, normalized to the reference genes elongation factor 1α (Ef-1α), β-2 microglobulin (B2m) and glyceraldehyde-3-phosphate dehydrogenase (Gapdh), were determined as described (Dietert et al. 2014). RT-qPCR and data analyses were conducted using the CFX96 Touch Real-Time PCR Detection System and CFX Manager software 1.6 (BioRad). Relative quantification and comparison of groups were performed by the ΔΔCt method using naive animals as controls.

### Histochemistry, immunohistochemistry and quantification of cells

For visualization of mucus cells, sections were processed as described and the PAS reaction was conducted (Leverkoehne and Gruber 2002). Immunohistochemical analyses were performed as described (Braun et al. 2010; Leverkoehne and Gruber 2002). Briefly, entire murine FFPE lungs with tracheas or FFPE tissue samples from humans and pigs were cut at 2 µm thickness and mounted on adhesive glass slides. After dewaxing in xylene and rehydration in decreasing ethanol concentrations, antigen retrieval was performed with 0.1 % protease pretreatment for 10 min at 37 °C (AppliChem, Darmstadt) for the detection of mCLCA5, mCLCA3 or club cell protein 10 and with microwave heating (600 W) in 10-mM citric acid (750 ml, pH 6.0) for 12 min for the detection of cytokeratin 5. For single stainings, slides of murine lungs were incubated with immunopurified rabbit antibodies for mCLCA5 (α-mCLCA5-C1-ap, 1:300) (Braun et al. 2010), mCLCA3 (α-m3-C-1p, 1:600) (Bothe et al. 2011) or cytokeratin 5 (1:1,000; ab24647, Abcam) or with the immunopurified goat antibody for club cell 10 protein (CC10, 1:1,500; sc-9772, Santa Cruz Biotechnology). To exclude cross-reactivity of the mCLCA5 antibody with other murine CLCA members, immunohistochemical stainings of the intestine, expressing mCLCA3, mCLCA4, mCLCA6 and mCLCA7 (Patel et al. 2009), and the pancreas, expressing mCLCA1/2 (Roussa et al. 2010), were performed and yielded negative results. Furthermore, specificity of the mCLCA5 antibody was previously verified by immunoblot analysis (Braun et al. 2010). Slides of porcine lungs were incubated with the immunopurified rabbit antibody for pCLCA2 (p2-C-1a, 1:300) (Plog et al. 2012b). The human lung sections were incubated at 4 °C over night with the pCLCA2 antibody which yielded an identical, specific, cellular staining pattern, pointing toward cross-reactivity with the human hCLCA2. Incubation with an immunopurified, irrelevant rabbit or goat antibody at similar dilutions served as negative control for all immunohistochemical stainings on murine, porcine and human tissue samples. The slides were incubated with biotinylated, secondary goat anti-rabbit IgG (1:200, BA 1000, Vector, Burlingame, CA) or rabbit anti-goat IgG (1:200, BA 5000, Vector, Burlingame, CA) antibodies and HRP- or AP-coupled streptavidin. Diaminobenzidine (DAB) or triamino-tritolyl-methanechloride (Neufuchsin) was used as substrates for color development, respectively. The slides were counterstained with hematoxylin or PAS reaction where indicated, dehydrated through graded ethanol, cleared in xylene and coverslipped.

**Fig. 1:**
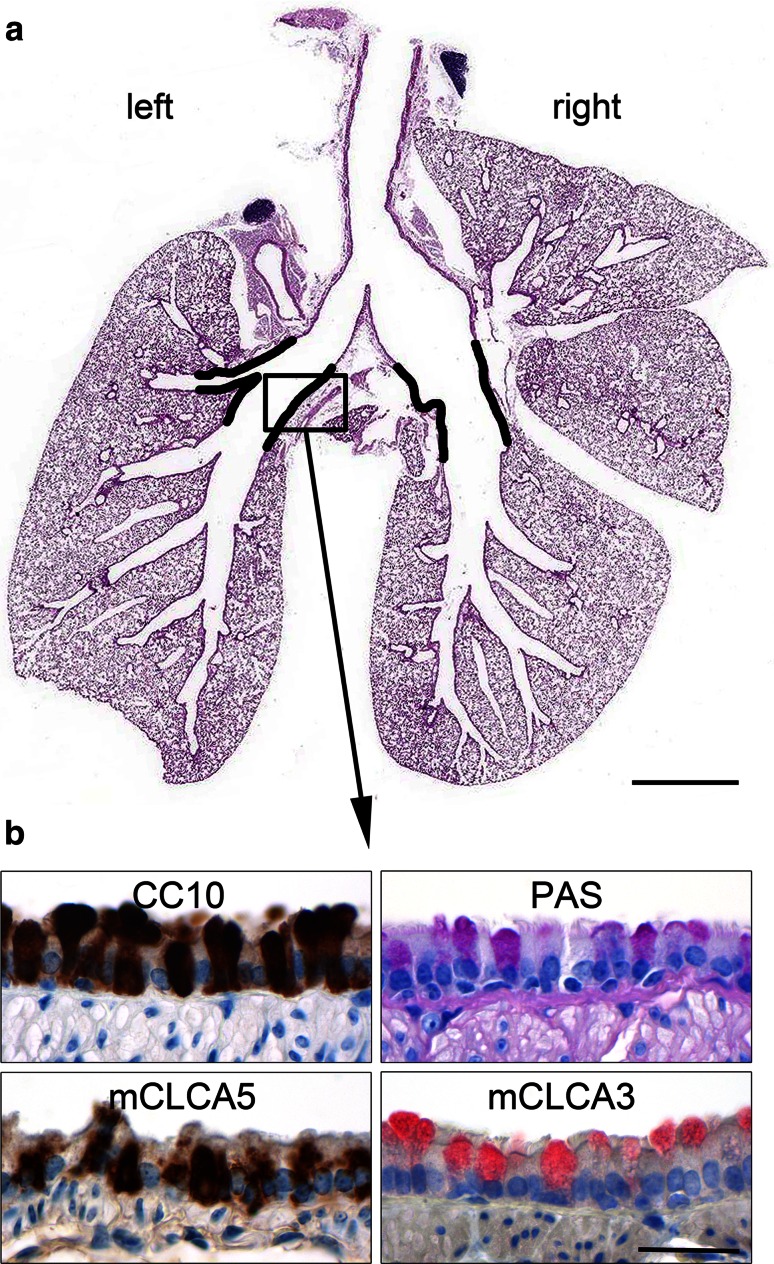
mCLCA5 is expressed in lungs of naive mice in a highly selective pattern. **a** Whole lungs of naive mice were embedded in paraffin, and multiple sections of defined layers were prepared to ensure that the entire bronchial stem and its branching points are available for systematic investigation. mCLCA5 protein is expressed in bronchial epithelial cells at the transition from the extrapulmonary main bronchi to the intrapulmonary bronchi (*black lines*) only. HE staining. **b** This region was characterized by CC10-positive club cells, PAS-positive mucus cells as well as mCLCA5- and mCLCA3-expressing cells. *Bar* (**a**) 2 mm, *bar* (**b**) 20 µm

Immunohistochemical double staining of mCLCA5 and mCLCA3 was performed using the H_2_O-elution method in accordance with the instructions of zytomed systems (Zytomed 2009). This method is suitable for double staining using primary antibodies from the same species. Therefore, slides were prepared as described above and incubated with the mCLCA5 antibody (1:300) at 4 °C over night. After incubation with the biotinylated, secondary goat anti-rabbit IgG antibody (1:200), DAB was used for color development. Due to using two secondary antibodies against the same species, unspecific binding was excluded by washing the slides in heated, deionized water (750 ml microwaved at 600 W for 10 min) to eliminate remaining unbound primary antibodies with a consecutive rinse in water at 4 °C for 5 min. Following incubation with the purified mCLCA3 antibody (1:600) at 4 °C over night and with the secondary, goat anti-rabbit IgG alkaline phosphatase-conjugated antibody (1:500, AP-1000, Vector, Burlingame, CA), triamino-tritolyl-methanechloride (Neufuchsin) was used as substrate for color development. Slides that were incubated with an irrelevant immunopurified rabbit antibody served as negative controls. To ensure specific binding of the secondary either HRP- or AP-conjugated antibody with the mCLCA5- or mCLCA3-specific primary antibody, respectively, slides were incubated with only one primary but with both secondary antibodies. Finally, slides were counterstained with hematoxylin, dehydrated, cleared and coverslipped. PAS-, mCLCA5-, mCLCA3- and CC10-positive cells were counted per millimeter of basement membrane at four anatomically defined regions of the bronchial epithelium at the extra- to intrapulmonary junction (Fig. 1, black lines) of naive, PBS-treated or *S. aureus*-infected mice using digital image software (AnalySIS docu 5.0., SIS).

### Immunofluorescence and spectral confocal laser scanning microscopy

For immunofluorescence co-localization analyses, slides were incubated with the purified, primary mCLCA5 antibody (1:50) over night at 4 °C as described above and with Alexa Fluor 488-conjugated, secondary donkey anti-rabbit IgG antibody (1:2,000, Invitrogen) for 1 h at room temperature. Slides were then incubated with the purified, primary CC10 antibody (1:50) at 4 °C over night, incubated with Alexa Fluor 594-conjugated, secondary donkey anti-goat IgG antibody (1:2,000, Invitrogen) for 1 h at room temperature and mounted with Roti-Mount FluorCare DAPI (4,6-diaminidino-2-phenylindole) (Carl Roth, Karlsruhe, Germany). Adequate negative controls, including incubation of slides with only one primary but both secondary antibodies, were conducted. Slides were analyzed by spectral confocal microscopy with a LSM 780 microscope (objective 40×, Plan-Neofluar/oil, NA 1.3; Zeiss, Jena, Germany).

### Data analysis

Data are expressed as mean ± SEM. Statistical analyses were performed using the Mann–Whitney test. *p* < 0.05 was considered significant.

## Results

### mCLCA5 is expressed in select bronchial epithelial cells at the transition from the extrapulmonary main bronchi to the intrapulmonary bronchi

For systematic expression analyses of mCLCA5 in the naive murine respiratory tract, trachea and entire lungs including bronchial stem and its branching points were investigated (Fig. 1a). The mCLCA5 protein was exclusively localized in bronchial epithelial cells of a defined region of approximately 2 mm in length (black lines) at the extra- to intrapulmonary junction as well as in epithelial cells of the SMGs which are, in mice, restricted to the larynx and the proximal trachea. For quantification of the different cell types of the bronchial epithelium in this specific region, the numbers of mCLCA5-, mCLCA3-, CC10-positive cells as well as PAS-positive mucus cells per millimeter of basement membrane were determined (Fig. 1b). In this specific location, 70.9 ± 2.7 % of the bronchial epithelial cells were positive for the club cell marker CC10, followed by 51.5 ± 1.7 % of mCLCA5-positive cells, 23.2 ± 3.2 % of PAS-positive cells and 20.5 ± 2.9 % of mCLCA3-positive cells (mean ± SEM, *n* = 4). Cytokeratin 5 expressing basal cells was localized only in the tracheal epithelium as well as in the cartilaginous bronchial epithelium and was clearly absent from the regions that possess mCLCA5-expressing cells (data not shown).

### mCLCA5 is predominantly located in club cells and, to a lesser extent, in ciliated cells and mucus cells of the bronchial epithelium, with a cell type-specific, subcellular expression pattern

**Fig. 2:**
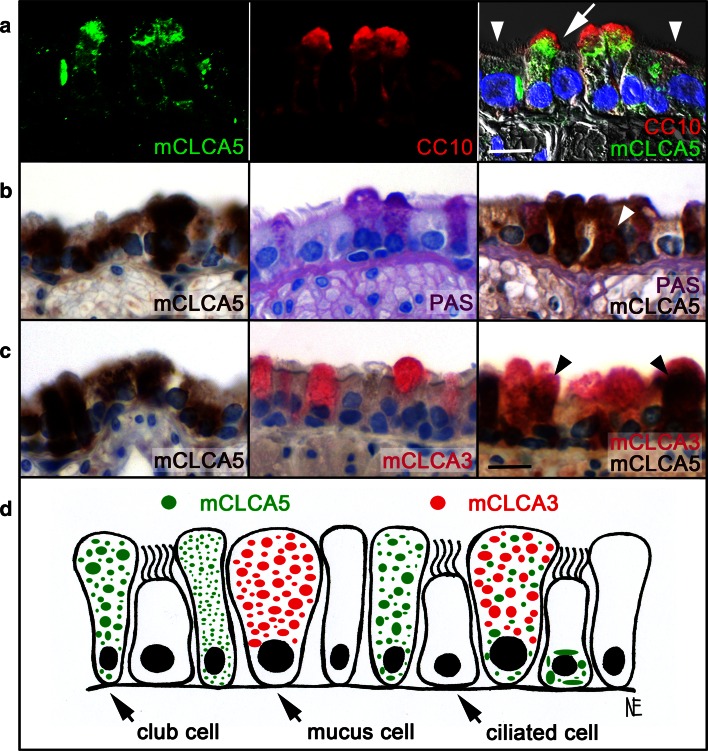
mCLCA5 is predominantly located in club cells, to lesser extent in mucus cells and ciliated cells. **a** For co-localization studies of mCLCA5 and club cell protein CC10, immunofluorescence and spectral confocal laser scanning microscopy was performed. (mCLCA5: *left*, *green*; CC10: *center*, *red*; merged image: *right*). *Blue* DAPI (4,6-diaminidino-2-phenylindole) staining of the DNA in the nuclei. **b**, **c** Double staining of mCLCA5 either with PAS reaction, identifying mucus cells, or with mCLCA3 by immunohistochemistry was conducted. mCLCA5 is primarily located in club cells (**a** *arrow*), followed by fewer ciliated cells (**a** *arrowhead*) and mucus cells (**b** *arrowhead*). In mucus cells, mCLCA5 was occasionally co-localized with mCLCA3 (**c** *arrowhead*). **d** Club cells and mucus cells showed an either fine or coarse, diffuse, granular, cytoplasmatic, subcellular labeling pattern of mCLCA5, in contrast to ciliated cells, which displayed a clumpy and perinuclear labeling pattern. However, only a limited number of the investigated cells were positive for mCLCA5 protein expression. *Bar* (**a**) 5 µm, *bar* (**b**, **c**) 10 µm

For identification of mCLCA5-expressing cells, immunofluorescence and spectral confocal laser scanning microscopy was performed. mCLCA5 was predominantly expressed in CC10-positive club cells (Fig. 2a, arrow) with a diffuse, either finely or coarsely granular, cytoplasmic pattern. However, only a limited number of club cells were positive for mCLCA5 protein expression. The protein was also detected in a few ciliated cells (Fig. 2a, arrowhead), displaying a clumpy and perinuclear expression signal. Further co-localization studies revealed mCLCA5 expression in few mucus cells (Fig. 2b, arrowhead), occasionally co-localized with the mucus cell marker mCLCA3 (Fig. 2c, arrowhead, d).

### mCLCA5 mRNA and protein strongly decrease after various challenges

**Fig. 3:**
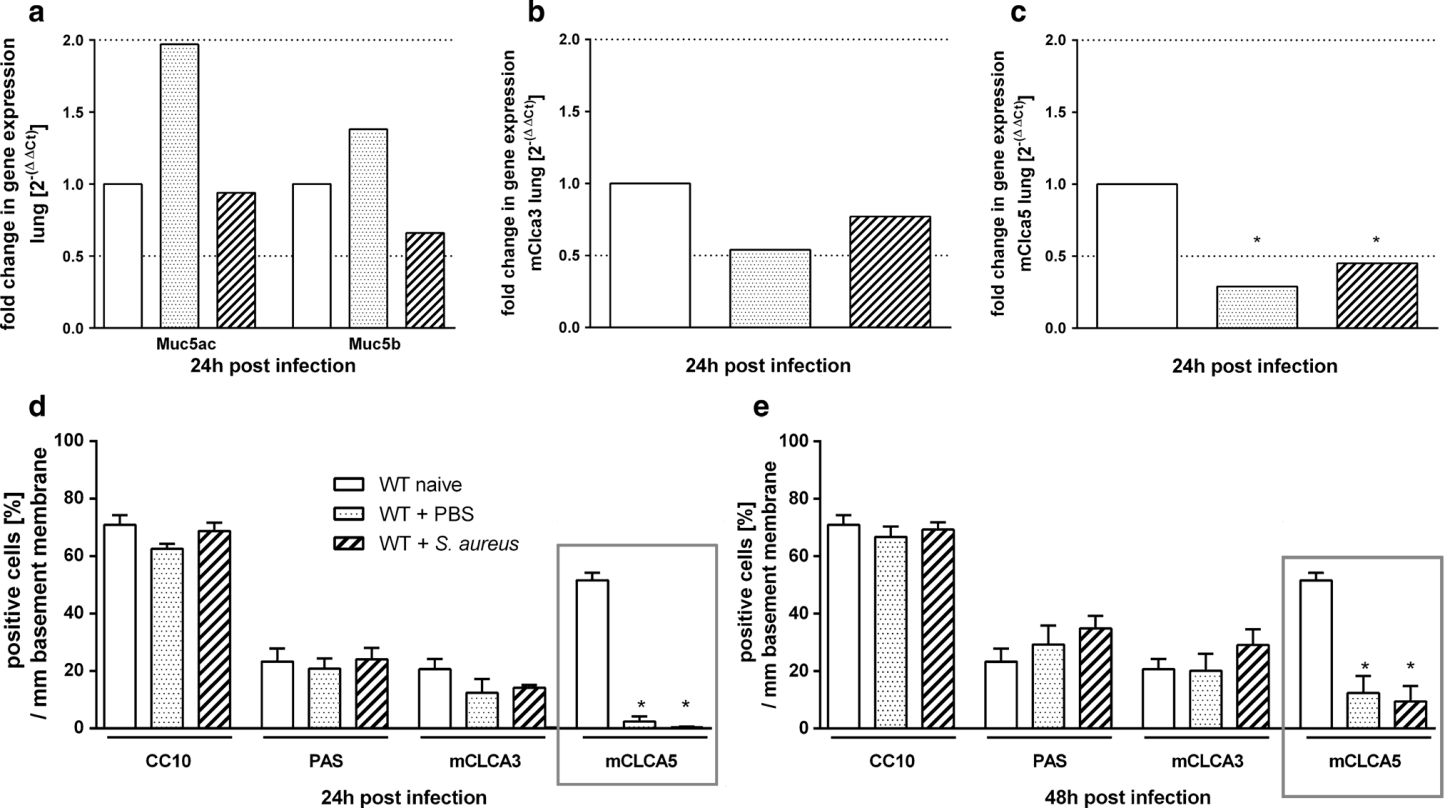
mCLCA5 mRNA and protein are strongly decreased in challenged lungs. **a**–**c** 24 h after mice were treated with PBS or infected with *S. aureus*, lung mRNA expression levels of Muc5ac, Muc5b, mClca3 and mClca5 were determined by RT-qPCR in comparison with naive mice. Only mClca5 mRNA was significantly decreased in both challenge models (**c**). *Dotted lines* indicate fold changes of 0.5 and 2, respectively, as limits for valid statement of lowered and elevated parameters. Values are given as mean ± SEM (*n* = 8 each group). *Ct* cycle threshold. *p < 0.05 versus the naive control group. **d**, **e** Numbers of CC10-, PAS-, mCLCA3- and mCLCA5-positive cells per mm basement membrane were quantified by immunohistochemistry or PAS reaction. mCLCA5-positive cells were significantly reduced in both challenge models at indicated time points, without any further changes in number or composition of the bronchial epithelial cells. Values are given as mean ± SEM (*n* = 4 each group). *p < 0.05 versus the naive control group

**Fig. 4:**
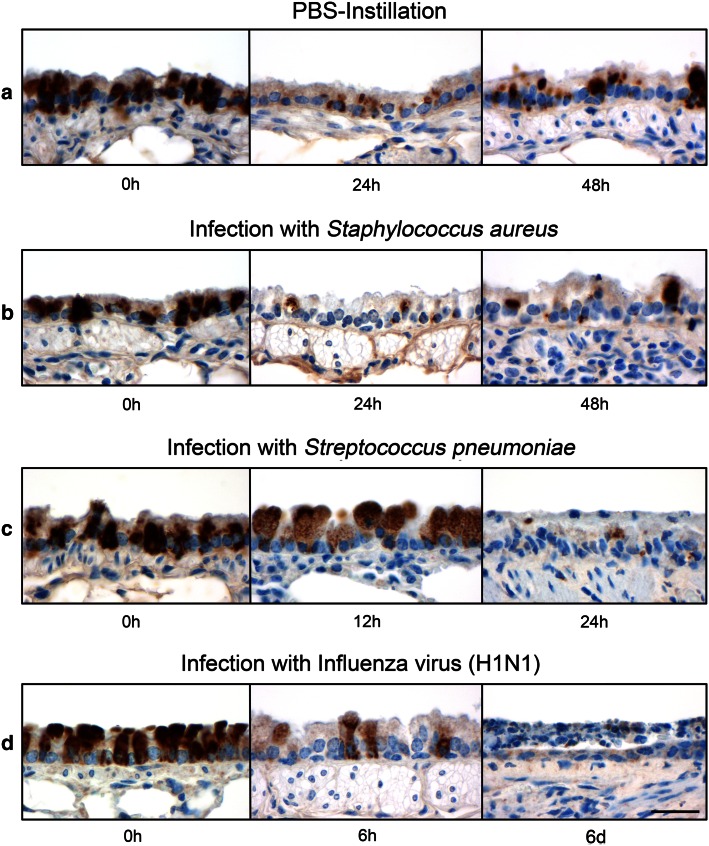
mCLCA5 protein expression disappeared in various challenge models. Lungs from naive (*n* = 4) and PBS-treated (*n* = 4) mice as well as from mice infected with *S. aureus* (*n* = 4), *S. pneumoniae* (*n* = 2) and influenza virus (*n* = 2) were examined at the extrapulmonary to intrapulmonary junction to characterize the presence and the course of mCLCA5 protein expression in this specific location at various time points. **a**, **b** Comparison of naive lungs to lungs from PBS-treated or *S. aureus*-infected mice revealed a significant reduction in mCLCA5 protein expression 24 h after infection, with a slight tendency toward a return after 48 h. **c**, **d** After infection with *S. pneumoniae* and influenza virus, the immunosignal of mCLCA5 disappeared over time. *Bar* 20 µm

mRNA levels of Muc5ac, Muc5b, mClca3 and mClca5 were quantified in lungs from naive, PBS-treated and *S. aureus*-infected mice by RTq-PCR. After 24 h, expression of both the mucin genes Muc5ac and Muc5b as well as of mClca3 was not altered compared to naive controls, independently of the type of challenge (Fig. 3a, b). Only mClca5 was significantly decreased on mRNA level after PBS treatment and *S. aureus* infection (Fig. 3c). Quantification of CC10-, PAS- and mCLCA3-positive cells per mm basement membrane revealed no differences between PBS-treated or *S. aureus*-infected mice compared to naive controls at all time points investigated (Fig. 3d, e). In contrast, mCLCA5-positive cells were significantly reduced 24 h after PBS treatment and *S. aureus* infection compared to naive mice (Figs. 3d, 4a, b). Despite this significant decrease which was still present after 48 h, the epithelium showed a slight tendency toward increasing numbers of mCLCA5-positive cells (Figs. 3e, 4b) which were significantly elevated (*p < 0.05) in *S. aureus*-infected mice. Additionally, after infection of mice with *S. pneumoniae* (Fig. 4c) or influenza virus, which both caused significant cell damage and loss in this area (Fig. 4d), a gradual reduction of mCLCA5-positive cells was observed over time without returning, possibly due to the initiated epithelial damage by these two pathogens.

### Human and porcine mCLCA5 orthologs are expressed in submucosal glands but not in bronchial epithelial cells

**Fig. 5:**
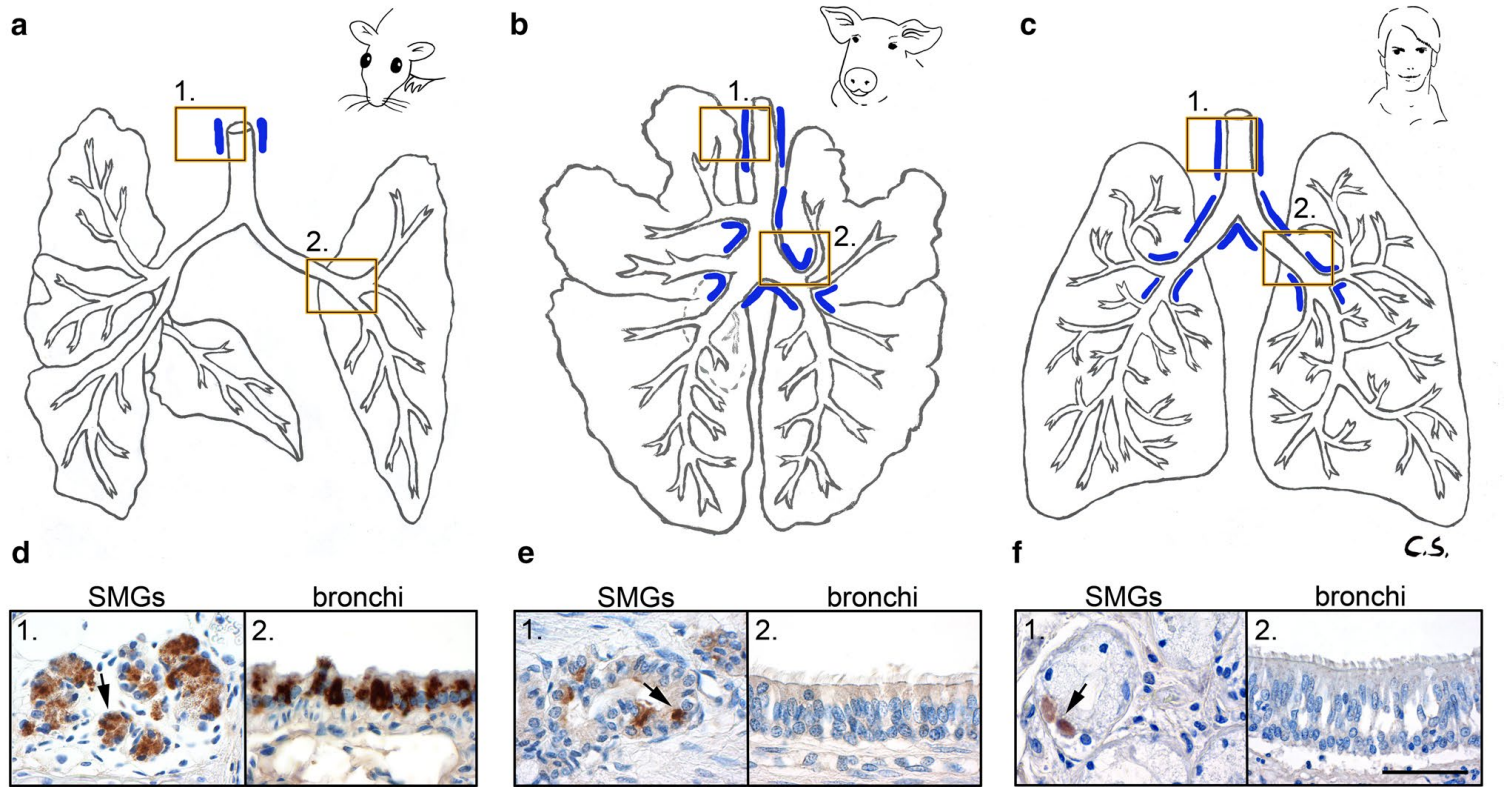
Species-specific differences in expression patterns of mCLCA5 and its human and porcine orthologs. Murine (*n* = 4), human (*n* = 2) and porcine (*n* = 3) lung tissues were investigated by immunohistochemistry. **a**–**c** A species-specific distribution pattern in the submucosal glands (*blue lines*) was observed, and all species investigated had mCLCA5-, hCLCA2- or pCLCA2-positive cells, respectively, in the epithelial cells of these SMGs (*d1*, *e1*, *f1*, *left picture*). However, only the mouse had mCLCA5-positive cells in this specific location within the bronchial epithelium (*d2*, *e2*, *f2*, *right picture*). *Bar* 40 µm

In order to determine possible species-specific differences as seen for other CLCA gene family members, the respiratory expression patterns of the mCLCA5 orthologs, hCLCA2 and pCLCA2, were immunohistochemically examined in human or porcine lungs, respectively. In mice, SMGs are only present in the upper part of the trachea (Fig. 5a, blue lines), whereas in the human and porcine respiratory tracts, these glands line the entire cartilaginous airways down to their branching into segmental bronchi (Fig. 5b, c, blue lines). The epithelial cells of these species-specifically distributed submucosal glands were positive for the respective CLCA orthologs in mice, humans and pigs in which the murine mCLCA5 signal was much stronger than in those of the respective orthologs (Fig. 5d–f, left picture). In contrast to the murine mCLCA5, neither its human nor its porcine ortholog was expressed in bronchial epithelial cells or other cell types throughout the entire lungs (Fig. 5d–f, right picture).

## Discussion

In the current study, we identified a unique mCLCA5 expression pattern in mouse airways which is restricted to two specific locations. On the one hand, mCLCA5 is expressed in the epithelial cells of the SMGs and, on the other hand, in the bronchial epithelium, specifically at the transition of the extrapulmonary main bronchi into the intrapulmonary bronchi. Interestingly, both regions are anatomically described as progenitor cell niches which have been characterized by several studies in detail (Liu and Engelhardt 2008; Rawlins and Hogan 2006; Roomans 2010; Warburton et al. 2008).

Club cells were the predominant cell type in the bronchial epithelia intensely expressing mCLCA5, whereas mucus cells and ciliated cells showed a reduced or absent expression of the mCLCA5 protein. Based on this apparently unique and highly specific distribution pattern of the mCLCA5 protein in airway epithelial cells, we can virtually exclude mCLCA5 protein expression in other known select, functionally distinctive airway epithelial cells with a characteristic tissue distribution. In particular, basal cells in the tracheal and cartilaginous bronchial epithelium as well as club cells located more distally from this location, and alveolar epithelial cells type II clearly do not express mCLCA5. In particular, a specific subset of club cells, the variant club cell type, which is primarily located in the non-cartilaginous bronchi and bronchioles (Liu and Engelhardt 2008; Rawlins and Hogan 2006; Roomans 2010; Warburton et al. 2008), is a known progenitor cell for non-ciliated club cells, ciliated cells and mucus cells (Pardo-Saganta et al. 2013; Rawlins and Hogan 2006; Rawlins et al. 2009; Reader et al. 2003; Reynolds and Malkinson 2010; Roomans 2010; Wong et al. 2009). A previous study identified mCLCA5 as being sufficient to induce mucus production and responsible for mucus cell metaplasia (Patel et al. 2009). Thus, the predominant expression of mCLCA5 in club cells as putative progenitors of mucus cells and its presence in known anatomical locations of progenitor cell niches would be in line with its proposed function in mucus cell differentiation. It is conceivable that mCLCA5 expression by precursor mucus cells drives their differentiation into mature mucus cells, as it has already been hypothesized for the closely related proteins hCLCA1 and mCLCA3 (Alevy et al. 2012; Patel et al. 2006, 2009). These findings were confirmed by the fact that a role in epithelial differentiation of other lineages has already been shown for several CLCA members (Alevy et al. 2012; Patel et al. 2006, 2009; Walia et al. 2012; Yu et al. 2013) and is also consistent with the suspected role of mCLCA5 in growth arrest and maturation processes of squamous epithelial cells of the skin (Beckley et al. 2004; Braun et al. 2010). The identified expressing cell types showed a distinct intracellular mCLCA5 protein distribution pattern. In club cells and in the few mucus cells, mCLCA5 displayed a diffuse, finely granular, cytoplasmatic pattern as well as an evenly distributed but coarse and clumpy pattern throughout the entire cytoplasm. In ciliated bronchial cells, a large, clumpy, perinuclear pattern dominated, suggesting that the subcellular protein distribution may depend on the differentiation status of the respective cell type or on a cell type-specific arrangement of organelles. Moreover, it is well conceivable that the mCLCA5 protein in mucus cells and ciliated cells is lost with progressing differentiation from airway precursor to mature cells.

Similar to mCLCA5, the murine mCLCA3 is a known inducer of mucus cell metaplasia (Patel et al. 2006) and it has been suspected that mCLCA5 and mCLCA3 may have redundant functions, with one compensating for the loss of the other (Patel et al. 2006, 2009). In our study, mCLCA5 and mCLCA3 had only partially overlapping expression patterns in mucus cells and mCLCA3 was neither expressed in club cells nor in ciliated cells (Leverkoehne and Gruber 2002). This may be suggestive of different functional relevance of these two related proteins in their respective cellular microenvironments.

Expression of mCLCA5 was further characterized in lung tissues that were available from previous challenge models (Dames et al. 2014; Dietert et al. 2014; Reppe et al. 2009). Under challenged conditions and independently of the type of challenge (PBS or *S. aureus*), mCLCA5 strongly decreased both on mRNA and protein levels at 24 h after challenge. Interestingly, after 48 h, the mCLCA5 protein signal reappears, possibly due to the epithelium remaining intact in these two models. While mCLCA5 mRNA expression and number of mCLCA5-positive cells decreased, the overall number and cell type composition of club cells and mucus cells, the major expressing cell types of mCLCA5, remained constant, pointing toward a selective transcriptional regulation of mCLCA5 with subsequent loss of the protein. Secretory processes by club cells and mucus cells which have been observed under challenged conditions (Davis and Dickey 2008; Evans et al. 2004; Pack et al. 1980; Reader et al. 2003; Reynolds and Malkinson 2010) may have contributed to the reduction of mCLCA5 on the protein level.

Under conditions of challenge, the downregulation of specific proteins involved in cellular differentiation is a known phenomenon of cells reacting to a specific type of challenge (Das et al. 2011; Zheng et al. 2013) which is consistent with our findings and the proposed role of mCLCA5 in cellular differentiation.

A similar effect on the mCLCA5 protein level was seen after infections with *S. pneumoniae* or influenza virus where a gradual reduction of mCLCA5-positive cells was observed over time without reappearance, possibly due to the initiated epithelial cell damage and death inflicted by these two pathogens. However, we cannot exclude that other, more specific factors may have contributed to the loss of mCLCA5 expression under the challenges used.

In a recent study comparing IL-13-challenged mice with PBS-treated controls, mCLCA5 protein was found in airway mucus cells, interpreted as a de novo expression (Mundhenk et al. 2012). However, mCLCA5 expression level and pattern in naive mice were not assessed in that study which would explain the fact that no differential upregulation of mCLCA5 mRNA was seen under challenged conditions (Mundhenk et al. 2012).

The murine lung, including the two niches that selectively express mCLCA5, differs from the lungs of other species in several anatomical and functional aspects. Murine SMGs are restricted to the larynx and the proximal trachea, whereas in humans and pigs, SMGs occur along the entire cartilaginous airways (Liu and Engelhardt 2008; Lynch and Engelhardt 2014; Rawlins and Hogan 2005; Rock et al. 2010; Suarez et al. 2012). The murine proximal airway epithelium predominantly consists of club cells, the principal secretory cell type (Liu et al. 2006; Rawlins and Hogan 2006; Reynolds and Malkinson 2010; Rock and Hogan 2011), followed by ciliated and fewer mucus cells (Pack et al. 1980; Wong et al. 2009). In contrast, ciliated and basal cells dominate in the human lung with much less secretory goblet cells (Rawlins and Hogan 2006; Rock et al. 2010; Wong et al. 2009). It is important to note that bronchial club cells, the major mCLCA5-expressing cell type in the mouse, do not exist in humans (Suarez et al. 2012). Furthermore, basal cells can only be found in the murine trachea and proximal cartilaginous airways (Rawlins and Hogan 2006; Rock et al. 2009), whereas in humans, they extend down to small bronchi (Fox 2007; Suarez et al. 2012; Wetzels et al. 1992). Based on these species-specific differences in airway anatomy and the suspected redundant functions of murine CLCA homologs (Patel et al. 2009), mice may not be the most suitable model for studying CLCA gene products in mucus cell metaplasia.

We therefore tested whether other species also express mCLCA5 orthologs in these specific niches of the respiratory tract. Specifically, we examined the protein expression patterns of hCLCA2 and pCLCA2, the direct orthologs to the murine mCLCA5, in human and porcine lungs. Interestingly, only very few human and some porcine SMG cells but no bronchial epithelial cells were found to express hCLCA2 or pCLCA2, respectively. It is tempting to speculate that this unique niche of mCLCA5-expressing cells in murine bronchial epithelium compensates for the lack of SMGs in the lower segments of murine airways.

The lack of hCLCA2 and pCLCA2 expression in the bronchial epithelium may point toward a species-specific function and is in line with the observation that hCLCA2 is not upregulated under mucus cell metaplasia, in contrast to its murine ortholog mCLCA5 (Alevy et al. 2012). Controversially, the similarity of mCLCA5 ortholog expression pattern between humans and pigs supports the notion that CLCA genes may be more closely related to the human than to the murine species as one would expect from the degree of sequence similarities (Plog et al. 2009, 2012a, b). The pig may thus become the preferred model in studying mucus cell metaplasia.

In summary, our results yielded several surprising observations on the distribution of mCLCA5 in the mouse lung and its human and porcine orthologs, hCLCA2 and pCLCA2. First, naive mice express mCLCA5 in very distinct niches of their bronchial epithelium and in epithelial cells of the SMGs. Second, under conditions of challenge, including instillation of PBS and infection with *S. aureus*, *S. pneumoniae* or influenza virus, mCLCA5 mRNA and protein expression strongly declined with protein reappearance only after challenges without epithelial cell damage. Third, the mCLCA5 orthologs, hCLCA2 and pCLCA2, are not expressed by bronchial epithelial cells in human and porcine lungs, respectively. Here, the orthologous proteins are present in SMG epithelial cells only, which, however, decorate the entire bronchial branchings. We speculate that the lack of these glands in most segments of the murine bronchial tree is compensated by additional mCLCA5 expression in a highly select area of the murine bronchial epithelium. Together with the results of previous studies on mCLCA5 and other CLCA homologs, our results raise several questions as to the role of these proteins in the maturation and differentiation of mucus cells. An approach including an ovalbumin challenge as the preferred model for studying mucus cell differentiation and mucus cell metaplasia in mice (Long et al. 2006; Nakanishi et al. 2001; Robichaud et al. 2005; Zhang and He 2010) may become of special interest in this issue.

## References

[CR2] Alevy YG (2012). IL-13-induced airway mucus production is attenuated by MAPK13 inhibition. J Clin Invest.

[CR3] Beckley JR, Pauli BU, Elble RC (2004). Re-expression of detachment-inducible chloride channel mCLCA5 suppresses growth of metastatic breast cancer cells. J Biol Chem.

[CR4] Bothe MK, Mundhenk L, Kaup M, Weise C, Gruber AD (2011). The murine goblet cell protein mCLCA3 is a zinc-dependent metalloprotease with autoproteolytic activity. Mol Cells.

[CR5] Braun J, Bothe MK, Mundhenk L, Beck CL, Gruber AD (2010). Murine mCLCA5 is expressed in granular layer keratinocytes of stratified epithelia. Histochem Cell Biol.

[CR6] Brouillard F (2005). Blue native/SDS-PAGE analysis reveals reduced expression of the mClCA3 protein in cystic fibrosis knock-out mice. Mol Cell Proteomics.

[CR7] Carter WG, Kaur P, Gil SG, Gahr PJ, Wayner EA (1990). Distinct functions for integrins alpha 3 beta 1 in focal adhesions and alpha 6 beta 4/bullous pemphigoid antigen in a new stable anchoring contact (SAC) of keratinocytes: relation to hemidesmosomes. J Cell Biol.

[CR8] Connon CJ, Yamasaki K, Kawasaki S, Quantock AJ, Koizumi N, Kinoshita S (2004). Calcium-activated chloride channel-2 in human epithelia. J Histochem Cytochem.

[CR9] Connon CJ, Kawasaki S, Yamasaki K, Quantock AJ, Kinoshita S (2005). The quantification of hCLCA2 and colocalisation with integrin beta4 in stratified human epithelia. Acta Histochem.

[CR1] Dames C et al (2014) Miniaturized bronchoscopy enables for unilateral investigation, application and sampling in mice. Am J Respir Cell Mol Biol. doi:10.1165/rcmb.2014-0052MA 10.1165/rcmb.2014-0052MA 24960575

[CR10] Das A, Acharya S, Gottipati KR, McKnight JB, Chandru H, Alcorn JL, Boggaram V (2011). Thyroid transcription factor-1 (TTF-1) gene: identification of ZBP-89, Sp1, and TTF-1 sites in the promoter and regulation by TNF-alpha in lung epithelial cells. Am J Physiol Lung Cell Mol Physiol.

[CR11] Davis CW, Dickey BE (2008). Regulated airway goblet cell mucin secretion. Annu Rev Physiol.

[CR12] Dietert K, Reppe K, Mundhenk L, Witzenrath M, Gruber AD (2014). mCLCA3 modulates IL-17 and CXCL-1 induction and leukocyte recruitment in murine *Staphylococcus aureus* pneumonia. PLoS One.

[CR13] Evans CM (2004). Mucin is produced by clara cells in the proximal airways of antigen-challenged mice. Am J Respir Cell Mol Biol.

[CR14] Fox J (2007). The mouse in biomedical research.

[CR15] Gibson A, Lewis AP, Affleck K, Aitken AJ, Meldrum E, Thompson N (2005). hCLCA1 and mCLCA3 are secreted non-integral membrane proteins and therefore are not ion channels. J Biol Chem.

[CR16] Hegab AE (2004). CLCA1 gene polymorphisms in chronic obstructive pulmonary disease. J Med Genet.

[CR17] Kamada F (2004). Association of the hCLCA1 gene with childhood and adult asthma. Genes Immun.

[CR18] Leverkoehne I, Gruber AD (2002). The murine mCLCA3 (alias gob-5) protein is located in the mucin granule membranes of intestinal, respiratory, and uterine goblet cells. J Histochem Cytochem.

[CR19] Liu X, Engelhardt JF (2008). The glandular stem/progenitor cell niche in airway development and repair. Proc Am Thorac Soc.

[CR20] Liu X, Driskell RR, Engelhardt JF (2006). Stem cells in the lung. Methods Enzymol.

[CR21] Long AJ, Sypek JP, Askew R, Fish SC, Mason LE, Williams CM, Goldman SJ (2006). Gob-5 contributes to goblet cell hyperplasia and modulates pulmonary tissue inflammation. Am J Respir Cell Mol Biol.

[CR22] Lynch TJ, Engelhardt JF (2014). Progenitor cells in proximal airway epithelial development and regeneration. J Cell Biochem.

[CR23] Mundhenk L (2012). mCLCA3 does not contribute to calcium-activated chloride conductance in murine airways. Am J Respir Cell Mol Biol.

[CR24] Nakanishi A (2001). Role of gob-5 in mucus overproduction and airway hyperresponsiveness in asthma. Proc Natl Acad Sci USA.

[CR25] Pack RJ, Al-Ugaily LH, Morris G, Widdicombe JG (1980). The distribution and structure of cells in the tracheal epithelium of the mouse. Cell Tissue Res.

[CR26] Pardo-Saganta A, Law BM, Gonzalez-Celeiro M, Vinarsky V, Rajagopal J (2013). Ciliated cells of pseudostratified airway epithelium do not become mucous cells after ovalbumin challenge. Am J Respir Cell Mol Biol.

[CR27] Patel AC (2006). Genetic segregation of airway disease traits despite redundancy of calcium-activated chloride channel family members. Physiol Genomics.

[CR28] Patel AC, Brett TJ, Holtzman MJ (2009). The role of CLCA proteins in inflammatory airway disease. Annu Rev Physiol.

[CR29] Plog S, Mundhenk L, Klymiuk N, Gruber AD (2009). Genomic, tissue expression, and protein characterization of pCLCA1, a putative modulator of cystic fibrosis in the pig. J Histochem Cytochem.

[CR30] Plog S, Grotzsch T, Klymiuk N, Kobalz U, Gruber AD, Mundhenk L (2012). The porcine chloride channel calcium-activated family member pCLCA4a mirrors lung expression of the human hCLCA4. J Histochem Cytochem.

[CR31] Plog S, Mundhenk L, Langbein L, Gruber AD (2012). Synthesis of porcine pCLCA2 protein during late differentiation of keratinocytes of epidermis and hair follicle inner root sheath. Cell Tissue Res.

[CR32] Rawlins EL, Hogan BL (2005). Intercellular growth factor signaling and the development of mouse tracheal submucosal glands. Dev Dyn.

[CR33] Rawlins EL, Hogan BL (2006). Epithelial stem cells of the lung: privileged few or opportunities for many?. Development.

[CR34] Rawlins EL (2009). The role of Scgb1a1+ Clara cells in the long-term maintenance and repair of lung airway, but not alveolar, epithelium. Cell Stem Cell.

[CR35] Reader JR, Tepper JS, Schelegle ES, Aldrich MC, Putney LF, Pfeiffer JW, Hyde DM (2003). Pathogenesis of mucous cell metaplasia in a murine asthma model. Am J Pathol.

[CR36] Reppe K (2009). Immunostimulation with macrophage-activating lipopeptide-2 increased survival in murine pneumonia. Am J Respir Cell Mol Biol.

[CR37] Reynolds SD, Malkinson AM (2010). Clara cell: progenitor for the bronchiolar epithelium. Int J Biochem Cell Biol.

[CR38] Robichaud A (2005). Gob-5 is not essential for mucus overproduction in preclinical murine models of allergic asthma. Am J Respir Cell Mol Biol.

[CR39] Rock JR, Hogan BL (2011). Epithelial progenitor cells in lung development, maintenance, repair, and disease. Annu Rev Cell Dev Biol.

[CR40] Rock JR (2009). Basal cells as stem cells of the mouse trachea and human airway epithelium. Proc Natl Acad Sci USA.

[CR41] Rock JR, Randell SH, Hogan BL (2010). Airway basal stem cells: a perspective on their roles in epithelial homeostasis and remodeling. Dis Model Mech.

[CR42] Roomans GM (2010). Tissue engineering and the use of stem/progenitor cells for airway epithelium repair. Eur Cell Mater.

[CR43] Roussa E, Wittschen P, Wolff NA, Torchalski B, Gruber AD, Thevenod F (2010). Cellular distribution and subcellular localization of mCLCA1/2 in murine gastrointestinal epithelia. J Histochem Cytochem.

[CR44] Suarez CJ, Dintzis SM, Frevert CW (2012) Respiratory. In: Elsevier (ed) Comparative anatomy and histology. Elsevier, Amsterdam, pp 121–134

[CR45] Walia V (2012). Loss of breast epithelial marker hCLCA2 promotes epithelial-to-mesenchymal transition and indicates higher risk of metastasis. Oncogene.

[CR46] Warburton D, Perin L, Defilippo R, Bellusci S, Shi W, Driscoll B (2008). Stem/progenitor cells in lung development, injury repair, and regeneration. Proc Am Thorac Soc.

[CR47] Wetzels RH (1992). Laminin and type VII collagen distribution in different types of human lung carcinoma: correlation with expression of keratins 14, 16, 17 and 18. Histopathology.

[CR48] Wong AP, Keating A, Waddell TK (2009). Airway regeneration: the role of the Clara cell secretory protein and the cells that express it. Cytotherapy.

[CR49] Yu Y, Walia V, Elble RC (2013). Loss of CLCA4 promotes epithelial-to-mesenchymal transition in breast cancer cells. PLoS One.

[CR50] Zhang HL, He L (2010). Overexpression of mclca3 in airway epithelium of asthmatic murine models with airway inflammation. Chin Med J (Engl).

[CR51] Zheng D, Limmon GV, Yin L, Leung NH, Yu H, Chow VT, Chen J (2013). A cellular pathway involved in Clara cell to alveolar type II cell differentiation after severe lung injury. PLoS One.

[CR52] Zytomed Systems (2009) Immunohistochemical double staining with highest flexibility. http://www.zytomed-systems.de/downloads-infomaterial/zytomed-systems-newsletter/9-9-newsletter-ausgabe-03-2009/file.html. Accessed 27 Aug 2014 (in German)

